# Construct of the Association between Sleep Quality and Perinatal Depression: A Literature Review

**DOI:** 10.3390/healthcare10071156

**Published:** 2022-06-21

**Authors:** Ana Filipa Poeira, Maria Otília Zangão

**Affiliations:** 1 School of Health, Polytechnic Institute of Setúbal, 2914-503 Setúbal, Portugal; ana.poeira@ess.ips.pt; 2 Comprehensive Health Research Centre [CHRC], Department of Nursing, Higher School of Nursing, University of Évora, 7000-811 Évora, Portugal

**Keywords:** perinatal care, depression, sleep hygiene, health promotion

## Abstract

Pregnancy is characterized by hormonal and physiological changes; some of these changes cause changes in sleep, presenting excessive sleep in early pregnancy due to the action of progesterone, and difficulty sleeping at the end of pregnancy due to weight gain and frequency of urination. Objective: to identify and systematize the evidence on the association between sleep quality and perinatal depression in pregnant and postpartum women. Methods: an integrative literature review was carried out with a search in the CINAHL, MEDLINE, and SCOPUS databases using the PRISMA flowchart. Results: Of the 92 articles, 10 studies were included according to the eligibility criteria. Results indicate that poor sleep quality during pregnancy is predictive of prenatal and postnatal depression. Sleep quality worsens with increasing gestational and maternal age. Conclusions: Sleep quality during pregnancy is associated with perinatal depression, a global public health problem with high prevalence. Due to its severe consequences for women, children, and families, perinatal depression needs to be identified early, preferably during pregnancy or soon after childbirth, justifying the priority of screening and prevention.

## 1. Introduction

Perinatal depression is a global public health problem with an estimated prevalence of 11.9% (95% CI, 11.4–12.5) according to a metaregression that included 96 studies [1]. Although the Diagnostic and Statistical Manual of Mental Disorders (DSM-5) requires that the specifier “peripartum” in depression be necessary for depression to occur during pregnancy or in the first four weeks postpartum, most experts in the field still define postpartum depression as occurring at any time in the first postpartum year regardless of the time of onset [2]. Thus, perinatal depression is defined as depressive symptoms that occur during pregnancy and those that continue or start in the first year postpartum [2]. Depression in pregnancy is highly likely to persist after childbirth if it is not diagnosed and treated on time [3]. Mood changes characterize this depressive disorder, decreased self-esteem, concentration, energy, increased tension, agitation, pessimism, guilt, ideas of self-mutilation, sleep disturbances, and weight changes [4]. Perinatal depression profoundly impacts the mother, child, and the rest of the family. For example, it has a negative effect on the child’s neurocognitive development, mainly when maternal depression occurs during the first year of life [5]. In addition, the parents’ experience causes family fractures and frustration related to what they consider to be the ideal paternity [4]. Clinically, sleep quality may be associated with an increased risk of perinatal depression [6]. Poor sleep quality is frequent during the prenatal period. In a study with 2427 pregnant women that aimed to characterize their sleep patterns and sleep problems in all months of their pregnancy, 76% of the women had poor sleep quality during pregnancy [7]. However, because sleep difficulty is assumed to be a common and temporary complication in pregnancy, few studies have effectively investigated sleep quality during pregnancy and its consequences. Thus, the objective of the present review is to identify and systematize the evidence on the association between sleep quality and perinatal depression in pregnant and postpartum women.

## 2. Materials and Methods

The integrative literature review can contribute to the discipline of nursing, namely, to the development of theory and with direct applicability to health practice and policies. It enables the synthesis of generalized knowledge on a given topic, presenting different perspectives and consequently identifying knowledge gaps [8]. Thus, the integrative literature aims to synthesize the results of research or theory using narrative analysis [9]. Several methods have been developed to elaborate literature reviews, and they continue to evolve rigor to the complexity of conducting an exhaustive and complete review. Integrative reviews allow for broader research, with an assortment of purposes and the potential to comprehensively portray complex concepts, theories, or health problems relevant to Nursing [8]. This review was based on the methodology of Whittemore and Knafl [8], which is structured as follows: problem identification; elaboration of the review question, definition of the research strategy with the description of inclusion and exclusion criteria for studies, categorization of studies to be included in the review, data analysis, and knowledge synthesis. To synthesize the knowledge of this review, narrative analysis was performed [9].

### 2.1. Review Question

Considering the theme, the following review question was defined: what is the association between sleep quality and perinatal depression (O) in pregnant women and women in the postpartum period (P)?

Adapted from the PICO acronym, we used PO: P = population—pregnant women and women in the postpartum period; O = phenomenon outcome—sleep quality and perinatal depression.

### 2.2. Inclusion and Exclusion Criteria

The inclusion criteria of the studies were: (1) studies that included pregnant participants and women in the postpartum period; (2) studies that included the sleep quality result measured by the Pittsburgh Sleep Quality Index and the assessment of depressive symptoms measured by the Edinburgh Postpartum Depression Scale, also admitting studies that included other measurement instruments as long as at least one of those was the Pittsburgh Sleep Quality Index or the Edinburgh Postpartum Depression Scale. As exclusion criteria, we defined: (1) studies whose participants were pregnant women with a depressive illness diagnosed before pregnancy, and (2) studies in which the exposure of interest was sleep respiratory pathology.

### 2.3. Search Strategy

A three-phase search was carried out: (1) a comprehensive and exploratory search on PubMed to identify evidence on the subject and consequently identify the most used terms for the further development of the search strategy; (2) an exhaustive search in the following databases (via the EBSCO service): Cumulative Index to Nursing and Allied Health Literature (CINAHL) Plus with Full Text, Medical Literature Analysis and Retrieval System Online (MEDLINE) with full Text, and SCOPUS; (3) the bibliographic references of the included studies were screened to identify possible studies of interest on athematic studies that were not screened in the performed search. The search formulas were adapted to each database as shown in Table 1. As limitations, we present the inclusion of studies that offered an available abstract in Portuguese and English, published between 2016 and 2021. and analyzed by peers.

### 2.4. Selection of Studies

**Figure 1:**
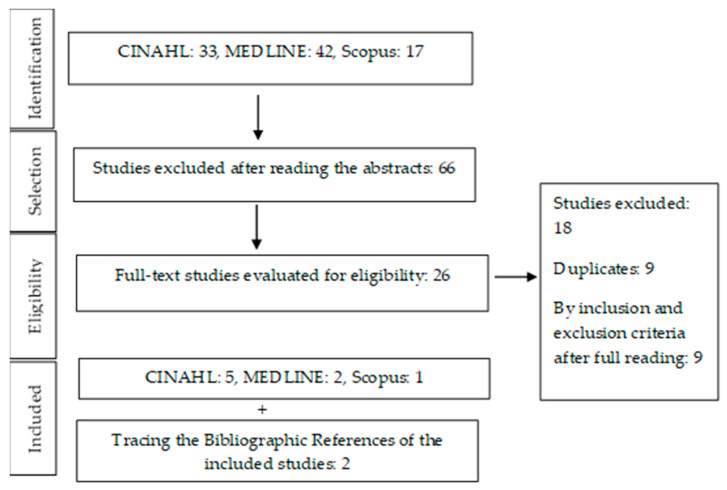
PRISMA flow diagram for selecting articles for review.

For the selection of studies, two reviewers read the titles and abstracts in an initial phase. These potentially relevant studies were then read in full, and selected according to the inclusion and exclusion criteria. In the absence of disagreement, including a third reviewer was unnecessary. The PRISMA flow diagram for determining articles for review is shown in Figure 1.

### 2.5. Data Extraction

For data extraction, an instrument was built that addressed the following topics: title, author, and year; periodical; type of study and level of evidence; objective; and main results. In interpreting the results, similarities were identified, and a synthesis of knowledge was carried out considering the main results of the included studies that answered the review question.

### 2.6. Methodological Quality Assessment

**Figure 2:**
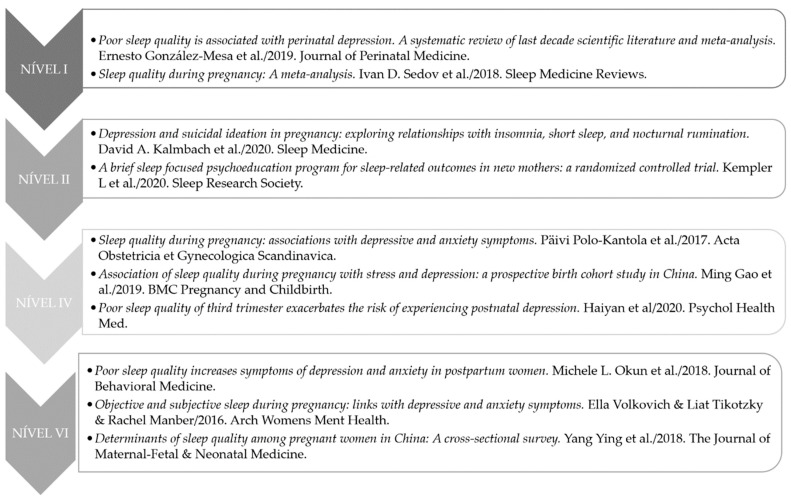
Included studies according to the hierarchical level of scientific evidence [11,12,13,14,15,16,17,18,19,20].

The selected articles were evaluated according to the level of evidence that they presented according to the hierarchical classification of evidence by Melnyk and Fineout-Overholt [10]: Level I—systematic review or meta-analysis; Level II—randomized controlled and well-delimited clinical trials; Level III—controlled clinical trials without randomization; Level IV—case–control studies and cohort studies; Level V—systematic, descriptive, and qualitative review studies; Level VI—descriptive or qualitative studies; Level VII—opinion of authorities and/or expert committee reports. Figure 2 shows the systematization of the included studies according to the hierarchical level of scientific evidence.

## 3. Results

The main results extracted from the included studies in this integrative literature review are presented in Table 2.

## 4. Discussion

One of the first questions that arise is the accuracy of the definition of sleep quality itself when assessing sleep quality. This is because there is, in fact, a set of indicators of good sleep, that is, a group of objectively identifiable indicators of sleep characteristics underlying its quality. However, there are also subjective sleep quality factors that greatly influence individual sleep satisfaction [11]. The present review found that the most frequently used instrument to assess sleep quality is the Pittsburgh/Pittsburgh Sleep Quality Index. Quality Index (PSQI). However, it may be necessary for the pregnant population to adjust the previously validated cutoff score (≥5) for the classification of poor sleep quality, and a higher score may be necessary to differentiate those who need further assessment and intervention. The physiological changes of pregnancy causing difficulty in sleeping complicate determining what pregnant women should expect regarding the need for additional assessment and treatment [12].

Poor sleep quality was significantly associated with increased symptoms of depression and anxiety [18]. Studies show that sleep quality during pregnancy is associated with prenatal stress and depression [11,13,15,21]. Pregnancy is described as stressful for many women, and stress-related disorders such as insomnia and depression are highly prevalent in this period [13]. Although insomnia is considered to be an independent disorder, insomnia and depression are associated during the perinatal period [22].

Sleep quality worsens with increasing gestational and maternal age [15,16,20]. An experimental study carried out on 267 pregnant women found that pregnant women from the second half of their pregnancy onwards had higher levels of insomnia, nocturnal rumination, depression, and suicidal tendencies [13]. Sleep quality worsens as the pregnancy progresses, worsening in the last trimester [15,20]. In addition, the quality of sleep declines with increasing age [22]. In one study, pregnant women aged 30 and over were found to experience poorer sleep quality than that of pregnant women under 30. Pregnant women aged 30 or over are also more likely to experience stress and depressive symptoms during pregnancy, which probably increases the risk of postpartum depression [16].

Results indicate that poor sleep quality is expected during pregnancy [19] and may be a vital intervention target, as disturbed sleep is predictive of postpartum depression and sleep disturbances [12,23]. The meta-analysis that quantified the prevalence of poor sleep quality during pregnancy concluded that it is necessary to identify women who need treatment, and to develop and provide evidence on appropriate interventions [12]. The poor quality of prenatal sleep seems to be related to the poor quality of postnatal sleep, which can consequently increase depressive symptoms after childbirth [17]. However, few studies report on the potential role of postnatal sleep quality, and its relationship with prenatal sleep and perinatal depression [17]. Thus, future investigations should compare sleep patterns in pregnancy, and after childbirth and perinatal depression. Further studies are also necessary to understand the efficiency of exercise programs specialized for postpartum women who may be vulnerable to depression, since exercises such as Pilates improve the quality of sleep in pregnant women [24]. Women undergoing treatment for insomnia during the third trimester of pregnancy reported less symptomatology of postpartum depression than those who did not receive treatment, thus suggesting a link between sleep quality during pregnancy and perinatal depression [25]. There are also studies on mobile phone use as a strategy for treating perinatal depressive symptoms [26], which could be an advantage for rural areas with less access to health services.

Depressed pregnant women are not only underdiagnosed but also reluctant to seek help. So, it is essential to identify variables that may reveal prenatal symptoms of depression, and this can be an effective strategy to signal women who need additional care throughout the perinatal period [22]. In addition, prenatal depression is a significant risk factor for postpartum depression [27]. The association between poor sleep quality and perinatal depression leads to clinical complications, so specialist nurses in the field of maternal and obstetric health, and obstetricians should identify sleep quality in routine prenatal tests performed, thus avoiding the development of mood pathologies [11,14]. The American College of Obstetricians and Gynecologists (ACOG) recommends screening for depression and anxiety symptoms at least once during pregnancy and postpartum [28]. The literature shows that, as pregnancy progresses, sleep-related problems such as insomnia, daytime sleepiness, and poor sleep quality increase [29]. Pregnant women experience stress, anxiety, and depressive symptoms rising from 24 weeks to postpartum [30]. During regular prenatal care, signs of insomnia, difficulty in managing mental stress, and excessive preoccupation with pregnancy should be ruled out to reduce the rates of clinical depression and suicidal ideation [13]. Several studies presented the nonuse of objective measures to assess sleep quality as a limitation, so whenever possible, actigraphy should be included in the methodology of future studies [13,15,16,17,18]. The very meaning of sleep quality is still difficult to understand by subjective measures of women’s sleep quality, which often only reflect individual satisfaction with sleep [31].

**Figure 3:**
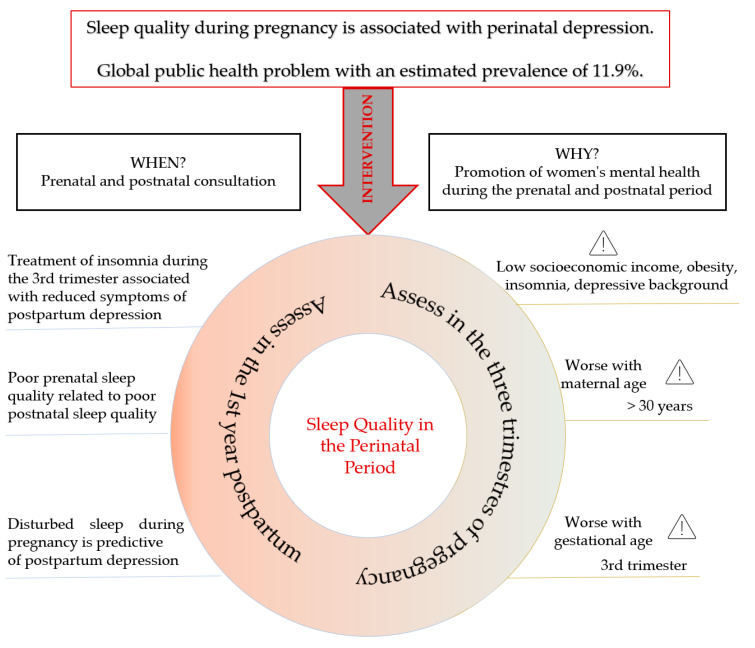
Construct of the synthesis of evidence on the association between sleep quality and perinatal depression.

The present review has limitations: most of the included studies were carried out in China, which could bias the results for the culture and practices in that region of the globe. Multiculturalism influences sleep patterns, so the particularities of the target population to whom care is provided should be considered when extrapolating the results of this review. Two secondary studies were included that summarize many primary studies on the subject. For this reason, the primary studies that constituted these meta-analyses were not included in this review. On the basis of the interpretation and synthesis of the identified evidence, a construct is presented with the particularities to be considered during prenatal and postnatal evaluation by health professionals in maternal and obstetric health (Figure 3).

## 5. Conclusions

Due to its severe consequences for women, children, and families, perinatal depression needs to be identified early, preferably during pregnancy or soon after childbirth, justifying the priority of screening and prevention. This review found that the issue of sleep quality has been explored for its relevance as a protective factor for mental health. Regarding health and the implementation of good practices in detecting signs and situations of risk, it is essential to emphasize the relevance of interventions that promote mental health in pregnancy. During pregnancy, poor sleep quality increases the risk of perinatal depression, even controlling for risk factors such as psychological distress and stress. Treatments for prenatal sleep should be explored, such as stress reduction using mindfulness and/or psychoeducational programs.

## Figures and Tables

**Table 1:** Databases and research strategy.

Database	Limiters	Search Strategy
CINAHL (EBSCOhost) (date of a most recent survey: 9 January 2022)	Full text; summary available; publication date: 1 January 2016–31 December 2021; English language; peer-reviewed. Expanders—apply equivalent subjects. Search Modes—Boolean/Phrase.	S11: S1 AND S9 AND S10 S10: S7 OR S8 S9: S2 OR S3 OR S4 OR S5 S8: AB Edinburgh postnatal depression scale OR AB EPDS S7: AB perinatal depression OR TI perinatal depression S6: AB PSQI OR AB Pittsburgh-sleep-quality-index S5: AB sleep-wake disorders OR TI sleep-wake disorders S4: AB sleep problem * OR TI sleep problem * S3: AB sleep quality OR TI sleep quality S2: (MH “Sleep”) S1: (MH “Pregnancy”)
MEDLINE with full text (EBSCOhost) (date of a most recent survey: 9 January 2022)	Full text; summary available; publication date: 1 January 2016–31 December 2021; English language; peer-reviewed. Expanders—apply equivalent subjects. Search Modes—Boolean/Phrase	S11: S1 AND S9 AND S10 S10: S7 OR S8 S9: S2 OR S3 OR S4 OR S5 S8: AB Edinburgh postnatal depression scale OR TI edinburgh postnatal depression scale S7: AB perinatal depression OR TI perinatal depression S6: AB (PSQI or Pittsburgh -sleep-quality-index) OR TI (PSQI or Pittsburgh-sleep-quality-index) S5: AB sleep-wake disorders OR TI sleep-wake disorders S4: AB sleep problem * OR TI sleep problem * S3: AB sleep quality OR TI sleep quality S2: (MH “sleep quality”) S1: (MH “pregnancy”)
SCOPUS (date of most recent search: 25 January 2022)	NA	(TITLE-ABS-KEY (sleep AND quality) AND TITLE-ABS-KEY (sleep AND problem) AND TITLE-ABS-KEY (perinatal AND depression)) AND PUBYEAR > 2016 AND PUBYEAR < 2022

NA, not applicable. * Truncation made the database return results that include any ending of that root word.

**Table 2:** Summary of the results of the included studies.

Author (Year), Periodical	Design	Data Collection Instruments	Study Population	Main Results *	Limitations *
Ernesto González-Mesa et al. [11] J Perinatal Med	Meta-analysis	NA	36 articles were included, of which eight were quantitative and used in meta-analysis	“(1) Both quantitative and qualitative reviews confirmed the negative relationship between the effects of poor sleep and perinatal mood changes, as demonstrated by the vast majority of studies, regardless of the objective or subjective assessment used. (2) It is not yet understood whether the links between poor sleep and perinatal depression are cause-effect or simply associative; therefore, more work is needed to address this gap in understanding the issue, including an investigation of the effects of medications and cognitive-behavioral therapies on sleep quality.”	“Heterogeneity of included studies with differences in the study designs: sample characteristics regarding cultural differences according to nationality, ethnicity or socioeconomic level, different selected assessment time points and different measurement tools.”
Ivan D. Senov et al. [12] Sleep Medicine Reviews	Meta-analysis	NA	Meta-analysis with 24 articles that measured the prevalence of sleep quality with the Pittsburgh Sleep Quality Index	“(1) The meta-analysis results indicate that the mean PSQI score during pregnancy is 6.07 and 45.7% of mothers experienced poor sleep quality as defined by a PSQI score ≥ 5. (2) A average PSQI score increased from the second to the third quarter by 1.68 points. (3) The cutoff score used to differentiate between good and bad sleepers suggested by the PSQI identifies the average number of pregnant women with poor sleep quality. (4) Future research should investigate longitudinal changes during sleep from preconception and throughout pregnancy to understand the relationship between sleep and gestational age.”	“Sleep poorly is still a reality during pregnancy, which complicates determining what pregnant women expect regarding the need for additional assessment and treatment. The previously validated cutoff score of five may be inappropriate for the pregnant population, and a higher score may be needed to differentiate those who need further assessment and intervention.”
David A. Kalmbach et al. [13] Sleep medicine	Randomized controlled trial	(1) Insomnia Severity Index (2) Edinburgh Postnatal Depression Scale (3) Presleep arousal scale cognitive	267 pregnant women in the 2nd and 3rd trimesters in a row at six hospitals in Detroit, USA	“(1) Women from the second half of pregnancy have bad insomnia, nocturnal rumination, depression, and suicidal tendencies. (2) Sociodemographic factors associated with higher rates of these complications during pregnancy include low economic income, absence of insurance, Black and non-Hispanic race, and obesity. (3) Clinical depression and suicidal ideation rates are higher for pregnant women with clinical levels of insomnia and high rumination. Rumination content may play an important role in disease risk, as pregnant women who ruminate specifically about their pregnancy and/or newborn have substantially higher depression and suicidal ideation rates than those of pregnant women who do not ruminate about perinatal concerns.”	“Only women identifying as non-Hispanic White and non-Hispanic Black were well-represented in this study, thus limiting generalizability to women outside of these racial groups. All sleep data were self-reported. There is no objective measurement of the sleep.”
Kempler L. et al. [14] Sleep Res Soc	Randomized controlled trial	(1)Pittsburgh Sleep Quality Index (2) Insomnia Severity Index (3) Multidimensional Assessment of Fatigue (4) Epworth Sleepiness Scale	215 healthy pregnant women in the 3rd trimester expecting their first child Sydney, Australia	“(1) Sleep quality declines for both groups at six weeks postpartum and with no statistically significant differences between groups. However, statistically, significant differences are observed between the 6th week and the 4th month postpartum, with the intervention group having the best sleep quality. The intervention consisted of a psychoeducation program. (2) The program proved to be simple, easy to adhere to, with low cost, and that it could easily be integrated into prenatal care.”	“It may be that the 4–6 month postpartum period is more sensitive to the effects of behavioral interventions. Due to the characteristics of the sample, it is not possible to generalize the results to a nonmetropolitan community setting. The sample elements with the poorest sleep during pregnancy were less likely to complete the study. Unselected women pregnant with their first child were recruited, which likely limited the scope for positive effects on some outcomes.”
Päivi Polo- Kantola et al. [15] Minutes Obstetrics et gynecological Scandinavian	Prospective cohort study	(1) Basic Nordic Sleep Questionnaire (2) Edinburgh Postnatal Depression Scale (3) State-Trait anxiety inventory	78 pregnant women with Gestational Age between 18 and 22 weeks of pregnancy at clinics in Turku, Finland	“(1) The quality of sleep generally declines as the pregnancy proceeds. (2) At the end of pregnancy, multiparous women reported greater difficulty falling asleep. (3) Insomnia and snoring get worse during pregnancy. (4) However, morning or daytime sleepiness and sleep duration did not increase despite sleep changes. (5) Results showed that mild to moderate levels of depressive and anxiety symptoms were related to sleep disturbances in late pregnancy.”	“Investigated self-reported sleep quality with no objective sleep measurements. It was not evaluated the occurrence of Willis–Ekbom Disease (Restless Legs Syndrome), which increases during pregnancy and may account for at least partially for sleep disturbances.”
Ming Gao et al. [16] BMC Pregnancy and Childbirth	Prospective cohort study	(1) Pittsburgh Sleep Quality Index pregnancy (2) Pressure Scale Edinburgh Postnatal (3) Depression scale	1152 2nd trimester pregnant women in a row at 54 hospitals and community health centers in urban areas of Shenyang, China	“(1) The linear regression model demonstrates that participants with poor sleep quality are more likely to experience stress during pregnancy, prenatal depression, and postnatal depression than participants with good sleep quality are. (2) Pregnant women aged 30 and over are at increased risk of prenatal stress and depression. (3) The association between sleep quality and postnatal depression was statistically significant in the group of women aged 30 years and over.”	“There may be residual confounders that were not taken into account. The findings are based on a regional population and may not generalize to other settings. There is no objective measurement of sleep (actigraphy).”
Haiyan et al. [17] Psychol Health Med	Prospective cohort study	(1) Pittsburgh Sleep Quality Index (2) 10-item Kessler psychological distress scale (3) Perceived stress scale (4) Edinburgh Postnatal Depression Scale	228 pregnant women in the 3rd trimester from the obstetrics and gynecology outpatient clinics of two hospitals in Shandong province, China	“(1) One month after delivery, 26.3% of women suffered from perinatal depression. (2) Univariate analysis of sociodemographic factors showed that postpartum depression was significantly associated with age and traumatic experience (e.g., death of a partner, divorce, financial bankruptcy). In addition, younger participants who had traumatic experiences in the past six months were more likely to have symptoms of depression. (3) Logistic regression analysis showed that poor sleep quality during pregnancy is associated with perinatal depression, regardless of risk factors such as psychological distress and stress.”	“It was used as a convenience sample; consequently, generalization of these findings maybe limited. Only used self-reported subjective measures.”
Michele L. et al. [18] J Behav Med	Cross-sectional	(1) Pittsburgh sleep quality index (2) Edinburgh postnatal depression scale (3) Overall Anxiety Severity and Impairment Scale (4) Patient Health Questionnaire -9-item	116 pregnant and postpartum women, Los Angeles, USA	“(1) Poor sleep quality was significantly associated with increased symptoms of depression and anxiety. (2) Sleep quality is a risk factor to affect the postpartum period. (3) Assessment of sleep hygiene during motherhood is worth considering as a component to identify women at risk for postpartum depression and anxiety.”	“This is a preliminary report of sleep quality measured only once. Sleep is recognized to change and often worsen across the perinatal period. There is no objective measurement of sleep (actigraphy).”
Ella Volkovich et al. [19] Arch Women’s Ment Health	Prospective cohort study	(1) Pittsburgh sleep quality index (2) Edinburgh postnatal depression scale (3) Beck anxiety inventory	148 pregnant women in the 3rd trimester expecting their first child, Helsinki, Finland	“(1) 50.3% of pregnant women reported poor sleep quality (defined as overall PSQI > 5). (2) 45% of women were awake on average for more than 30 min at night, and 27% had more than three night-time wakes per night. (3) Emotional distress (i.e., anxiety and severity of depressive symptoms) was significantly associated with self-reported sleep disturbances. (4) More quantitative PSQI components, such as sleep efficiency, were not associated with depressive and anxiety symptoms in the present study. The fact that correlations between emotional distress and different aspects of sleep subjectivity, as assessed by the PSQI, had variability suggests that the results are not merely the result of a self-report bias but likely reflect the complexity of the relationships between sleep and sleep emotional distress in pregnant women.”	“The sample characteristics (e.g., medium to high socioeconomic status, expecting a first child, and relatively low incidence of women scoring in the clinical range of depression) limit the generalizability of the results. Results may also not generalize to earlier stages of pregnancy.”
Yang Ying et al. [20] The Journal of Maternal-Fetal and Neonatal Medicine	Cross-sectional	(1) Pittsburgh Sleep Quality Index (2) Edinburgh Postnatal Depression Scale (3) Multidimensional scale of perceived social support	500 pregnant women from the obstetrics and gynecology outpatient clinic of two teaching hospitals in central China	“(1) Pregnant women had worse sleep quality in the third trimester. (2) In addition, depressive symptoms increase with age and gestational age, which is why they are one of the determinants of sleep quality in pregnant women. (3) Recommendation that health professionals pay more attention to sleep problems and provide sleep counseling during antenatal examinations, particularly for pregnant women with depressive tendencies and advanced maternal age.”	They are not mentioned.

* In order to not bias the interpretation of results and limitations, we chose to cite the information contained in the studies. NA—not applicable.

## Data Availability

Not applicable.
